# Natural detoxification of antibiotics in the environment: A one health perspective

**DOI:** 10.3389/fmicb.2022.1062399

**Published:** 2022-11-24

**Authors:** Fernando Baquero, Teresa M. Coque, José-Luis Martínez

**Affiliations:** ^1^Division of Biology and Evolution of Microorganisms, Department of Microbiology, Ramón y Cajal Institute for Health Research (IRYCIS), Ramón y Cajal University Hospital, and Centro de Investigación Biomédica en Red, Epidemiología y Salud Pública (CIBERESP), Madrid, Spain; ^2^Division of Biology and Evolution of Microorganisms, Department of Microbiology, Ramón y Cajal Institute for Health Research (IRYCIS), Ramón y Cajal University Hospital, and Centro de Investigación Biomédica en Red, Enfermedades Infecciosas (CIBERINFECT), Madrid, Spain; ^3^Centro Nacional de Biotecnología, CSIC, Madrid, Spain

**Keywords:** antibiotic detoxification, antibiotics in soil and water, antibiotic resistance in the environment, adsorption and desorption, particles in the environment

## Abstract

The extended concept of one health integrates biological, geological, and chemical (bio-geo-chemical) components. Anthropogenic antibiotics are constantly and increasingly released into the soil and water environments. The fate of these drugs in the thin Earth space (“critical zone”) where the biosphere is placed determines the effect of antimicrobial agents on the microbiosphere, which can potentially alter the composition of the ecosystem and lead to the selection of antibiotic-resistant microorganisms including animal and human pathogens. However, soil and water environments are highly heterogeneous in their local composition; thus the permanence and activity of antibiotics. This is a case of “molecular ecology”: antibiotic molecules are adsorbed and eventually inactivated by interacting with biotic and abiotic molecules that are present at different concentrations in different places. There are poorly explored aspects of the pharmacodynamics (PD, biological action) and pharmacokinetics (PK, rates of decay) of antibiotics in water and soil environments. In this review, we explore the various biotic and abiotic factors contributing to antibiotic detoxification in the environment. These factors range from spontaneous degradation to the detoxifying effects produced by clay minerals (forming geochemical platforms with degradative reactions influenced by light, metals, or pH), charcoal, natural organic matter (including cellulose and chitin), biodegradation by bacterial populations and complex bacterial consortia (including “bacterial subsistence”; in other words, microbes taking antibiotics as nutrients), by planktonic microalgae, fungi, plant removal and degradation, or sequestration by living and dead cells (necrobiome detoxification). Many of these processes occur in particulated material where bacteria from various origins (microbiota coalescence) might also attach (microbiotic particles), thereby determining the antibiotic environmental PK/PD and influencing the local selection of antibiotic resistant bacteria. The exploration of this complex field requires a multidisciplinary effort in developing the molecular ecology of antibiotics, but could result in a much more precise determination of the one health hazards of antibiotic production and release.

## Introduction: Molecular ecology of antibiotics in the critical zone

The concept of “molecular ecology” was proposed in 1976 by the biochemist Carlos Asensio as a field of investigation of the fate and the interactions among molecules in a local or global chemosphere (Asensio, 1976). In this review, we consider the interactions between antibiotics and inorganic and organic molecules in the environment, and the potential consequences of these interactions on the “critical zone” of human health and welfare: the Earth’s thin microbiosphere (Brantley et al., 2007). One of these consequences is the emergence, selection, or spread of antibiotic resistant bacteria (Grenni et al., 2018). This ecological approach could help to predict the environmental effects of antimicrobial agents on the inorganic and organic composition of specific environments in a variety of conditions.

It has been estimated that at least one-half of all antibiotics used in human and animal diseases (including antibiotic production industry) and in farming activities are released into the water and soil environments (Fukahori et al., 2011; Dutta and Mala, 2020). There, they interact with a complex bio and chemosphere under the influence of environmental factors, such as light, temperature, and pH (Zhi et al., 2019). The fate of antibiotic activity in the environment, and therefore the impact produced on the ecosystem (Cycoń et al., 2019), is a result of geochemical and biological interactions including geo-and bio-adsorption, accumulation, and degradation; most of which remain under-investigated. For further reading, comprehensive reviews on antibiotics in the environment are available (Baquero et al., 2008; Kümmerer, 2009a,b; Sodhi et al., 2021). The fate and effects of drugs, including antibiotics, in the environment should be understood in a changing context: the global annual growth rate of the pharmaceutical industry is estimated to be 6.5% due to factors as age, life span expectancy, economic growth, intensified livestock practices, and exacerbation of diseases due to climate change (OECD, 2019 *Pharmaceutical residues in freshwater: hazards and policy responses.* *Global Chemicals Outlook*. United Nations Environment Programme, 2019).

## Natural degradation and mineralization of antimicrobial agents

Antibiotics lose their effects at varying rates due to spontaneous molecular alterations. The estimated half-life of antibiotics differs in relation with its concentration and the type (abiotic and biotic composition) of soil, and, certainly, also by sampling-analytical procedures (Parthasarathy et al., 2018). As an indication, fluoroquinolones have a longer half-life (more than 5 years), followed by macrolides (2–3 years), tetracyclines (2 years), sulfonamides (2–3 months), and beta-lactams (days; Cycoń et al., 2019). Even the more persistent antibiotics, such as the quinolones or macrolides, are degraded and mineralized over a long period of time (Topp et al., 2016). For beta-lactams, the cleavage readiness of their beta-lactam bonds in aqueous solution is dependent on the pH and on the chemical structure of the drug (Yamana and Tsuji, 1976). Antibiotics with a higher h-Woodward-Fieser value as a measure of chemical reactivity are more prone to hydrolysis; for example, carbapenems and clavulanate are easily decomposed and monobactams are less susceptible (Turner et al., 2022). The final result of these processes is mineralization (the transformation of antibiotics into inorganic forms), or their transformation into smaller, simpler, and inactive organic compounds if mineralization is not complete (Bridgham and Ye, 2013; Adeyemi et al., 2021).

Antibiotics may adsorb many organic and inorganic surfaces due to electrostatic interactions, π-π bonding, weak Van der Waal forces, H-bonding, and surface complexation (Mangla et al., 2022). Antibiotics have variable adsorption coefficients (Kds) to soil materials. Biosphere proteins are ubiquitous in the soil (mostly originating from dead cells), and protein-binding of some groups of antibiotics can detoxify them. Geogenic organic carbons, also from anthropogenic origins (e.g., biochar and graphite), and also bentonite, humic and fulvic substances (the final break-down constituents of the natural decay of plant and animal materials) and clay minerals adsorb antibiotics. Consequently, these substances can be used to remove antibiotics from the environment (Ahmed et al., 2015; Wang et al., 2018; Zethof et al., 2019). However, given that antibiotics accumulate within these compounds but are not degraded, this accumulation might alter the structure of the associated microbiomes. These and other aspects will be discussed in the following sections.

## Minerals and antibiotic detoxification and degradation

Colloidal soil particles such as clay minerals are frequent in the environment and they mainly form fine-grained sediments and rocks. They are an important component of soils and sediments from rivers, lakes, estuarine, delta, and oceans, which cover most of the Earth’s surface. Clay minerals consist of particulated hydrous aluminum phyllosilicates, with a characteristic stratified structure formed by sheets with varying topologies, typically tetrahedral, and octahedral sheets with a size slightly larger than a bacterial cell. Such structures act as “chemically active geochemical platforms” that influence bacterial metabolism (Rong et al., 2007), where inorganic (e.g., metals) and organic molecules (e.g., antibiotics) adsorb and interact. The adsorption rate can be high, with maximum adsorption capacities over 100 mg/g (Hacıosmanoğlu et al., 2022; Yin et al., 2022). Clay platforms located where water and light are available serve to accelerate processes influencing chemical modifications, including photochemical transformations of antibiotics, which can triple (in the case of tetracycline) the rate of modifications in pure water without colloids (Liu et al., 2019).

The mechanism involved in antibiotic degradation and detoxification in wet mineral clays mostly depends on oxidation and super-oxidation processes, which are accelerated by light and metals, in a pH-dependent process (Ahmad et al., 2021). Photolysis (also known as photodecomposition, photodissociation, or photodegradation) is modulated by the presence of dissolved inorganic (e.g., nitrates) and organic matter (e.g., humic acids; Andreozzi et al., 2003; Zhan et al., 2006). The net result is oxidative modification and degradation of the antibiotic chemical structure, which attacks the double bonds, aromatic rings, and functional groups essential for antibiotic activity. A key process in this catalytic degradation are the Fenton/Fenton-like reactions associated with the iron redox cycle, in which the antibiotic plus an hydroxyl radical gives rise to a middle product and OH-and ultimately CO_2_ and H_2_O (Jiang et al., 2022). Iron-rich minerals in the environments (such as biotite, Fe-smectite, jarosite, magnetite, pyrite, hematite, amphibole, and goethite) contribute to the antibiotics’ and other organic compounds’ mineralization processes, producing simpler organic compounds if mineralization is not complete (Bridgham and Ye, 2013; Meyer et al., 2015). Also, antibiotics adsorption and degradation due to hydroxides/oxides of Cu^2+^ and by Cu^+^ atomic species probably occurs in nature (Oliveira et al., 2018). Natural and human-produced (present and past) vegetation fires lead to a considerable increase of charcoal into soils (González-Pérez et al., 2004). Most probably, part of this charcoal could be naturally activated into highly porous charcoal, very efficient in adsorbing and inactivating antibiotics (Liao et al., 2013; Zhang et al., 2016).

## Antibiotic inactivation by natural-organic matter in water and soil environments

Organic matter (particulate or dissolved) from natural waters is photochemically reactive (Cottrell et al., 2013), being able to degrade antibiotics. Direct photodegradation occurs by sunlight absorption, and indirect photolysis involves reactions with reactive photo-induced species as singlet oxygen (^1^O_2_), hydroxyl radicals (HO^●^), and the triplet excited state of chromophoric dissolved organic matter (^3^CDOM*) formed in natural waters. Those photochemical effects have been detected in aminoglycosides (Li et al., 2016). This effect is complex; for example in tetracyclines indirect photolysis might be enhanced, but direct tetracycline photolysis (sunlight absorption) can be inhibited (Song et al., 2021). The effects are highly dependent on factors such as pH and water depth (Lastre-Acosta et al., 2019).

Antibiotics absorb to natural polymers ubiquitous in the soil and water. Cellulose and chitin are the most abundant biopolymer polysaccharides in the environment. Cellulose exposed hydroxyl and reduced and nonreduced end groups, facilitating reactivity with pollutants, is mostly found in plant cell walls, but bacteria and algae also biosynthesize cellulose (Sayen et al., 2018; Tao et al., 2020; Juela, 2021). Decontamination preparations using cellulose derivatives adsorb a variety of antibiotics, such as tetracyclines, quinolones, sulfonamides, chloramphenicol, beta-lactams, and macrolides (in order from higher to lower absorption; Yao et al., 2017). Chitin is present in variety of soil and water invertebrates, usually in the surface exoskeleton of arthropods such as crustaceans, and in the cuticle or extracellular matrix of insects, fungi, sponges, mollusks, and nematodes. Chitin is a good adsorber of some antibiotic agents (Tunç et al., 2020). The fact that soil animals constitute about one-quarter of all animals on Earth is frequently overlooked, but it suggests that the influence of soil invertebrates might play a significant, largely ignored role in the fate of antibiotics and, in general, in the ecosystem (Lavelle et al., 2006; Zhu et al., 2019). Chitosan (deacetylated chitin) is not a known natural compound in the environment, but it can be used in environmental antibiotic de-contamination processes (Abd El-Monaem et al., 2022).

## Bacterial organisms and antibiotic biodegradation in the environment

One of the classic proposals regarding environmental effects and the natural degradation of antibiotics is the publication by Julian Davies, suggesting that antibiotic-producing microorganisms probably also contain mechanisms of antibiotic detoxification to avoid self-suicide of the population (Davies, 1994; Davies and Davies, 2010). Another possibility is that antibiotics could serve as weapons in “microbial wars,” essentially as defense mechanisms against competing organisms with antibiotic producers, to ensure permanence in their optimal niche. Antibiotic production is critical in sporulating microorganisms; their synthesis is triggered during the stationary phase of growth, which leads to spore formation. Ultimately, this energy-consuming process can require the degradation of mycelium (*Streptomyces*) or the mother cell (*Bacillus*; Yagüe et al., 2013; Roy et al., 2015). Since such self-nutrients should not be consumed by foreign microorganisms, such as bacteria and probably also Protozoa (Ahmetagic et al., 2011); the production of antibiotics against these competitors could prevent such consumption. If this hypothesis is true, a possible reaction of the potential invaders would be to biodegrade these inhibitory compounds. If antibiotic-producing microorganisms or widespread antibiotic resistant bacteria release a sufficient quantity of antibiotic-degrading molecules into the environment, this could impact the fate of antibiotics. However, the natural role of antibiotics in the environment could also be associated with cell-to cell communication; that is, “antibiotics as signaling agents” (Linares et al., 2006; Yim et al., 2006; Fajardo and Martínez, 2008; Aminov, 2009). By nature, signals should be ephemeral and should vanish after accomplishing their communication role. In fact, polymyxins (produced by Bacillaceae in relation with the sporulation process) are frequently hydrolyzed by *Bacillus* and *Paenibacillus*, but peptidases from Gram-negatives might also degrade these antibiotics (Yin et al., 2019).

The possibility of antibiotics serving as carbon or nitrogen nutrients cannot be ruled out. The term “antibiotic subsistence” was coined to refer to microbial organisms and communities subsisting on antibiotics (Dantas et al., 2008), a hypothesis suggested by Tony Medeiros in the 1990s (Medeiros, 1997). Several soil microorganisms, including *Pseudomonas* and *Burkhordelia* are able to grow on beta-lactams as a single carbon source (Jayaraman, 2009; Crofts et al., 2017). A later and broader study revealed that Burkholderiales, Pseudomonadales, Enterobacterales (mostly *Serratia*), Actinomycetales, Rhizobiales, and Sphingobacteriales from soil origin are able to subsist on antibiotics as a sole carbon source, and the spectrum of biodegraded antibiotics includes not only beta-lactams, but aminoglycosides, chloramphenicol, glycopeptides, quinolones and fluoroquinolones, sulphonamides, and trimethoprim (Dantas et al., 2008). These phenomena might also occur in the gut microbiota, and antibiotic-subsisting organisms from sewage communities might contribute to environmental antibiotic degradation, reducing selection for resistance (Perri et al., 2020; Deng et al., 2021; Lindell et al., 2022). A mechanism of extracellular molecular scavenging involving putrescine and lipocalins protects *Burkholderia cenocepacia* from bactericidal antibiotics, perhaps *via* an antioxidant effect (Naguib et al., 2022). All these functions imply that natural antibiotics should be present in the environment to fulfill ecological functions. However, as stated earlier, anthropogenic pollution with industrial antibiotics is a major source of antibiotics in the environment (Dantas et al., 2008).

At first glance, biotransformation of antibiotics with bacteria could represent a challenge due to the possible effect on biodegrading organisms (Olicón-Hernández et al., 2017). However, this effect is mitigated by non-microbial degradation and the typically low antibiotic concentrations in natural ecosystems (Bernier and Surette, 2013). In addition, antibiotic resistance could have evolved as a prior step to antibiotic catabolism with nutritional purposes, as can occur with aminoglycosides (de Bello González et al., 2015). On the other hand, we cannot rule out the possibility that biodegradative pathways with nutritional or signal-effacing purposes could be at the root of antibiotic resistance (Dantas et al., 2008; Lindell et al., 2022). Environmental microorganisms can degrade antibiotics in the environment by methyl-hydroxylation; aliphatic-aromatic rings hydroxylation; alcohols and amines oxidation; reduction of carboxyl groups; removal of methyl, carboxyl, fluoro, and cyano groups; addition of formyl, acetyl, nitrosyl, and cyclopentenone groups; opening aromatic rings, altering the loop structures, or removing functional chemical groups (Parshikov and Sutherland, 2012). For example, demethylations exerted by *Klebsiella* or *Stenothrophomonas maltophilia* can start the degradative process in tetracyclines (Leng et al., 2016; Ahmad et al., 2021). In the case of *Pseudomonas* and *Burkhordelia*, beta-lactam degradation occurs by co-expression of a β-lactamase, amidase, and up-regulation of phenylacetic acid catabolon (Crofts et al., 2018). *Klebsiella pneumoniae and Proteus mirabilis* could degrade ciprofloxacin *in vitro* by using mechanisms of hydroxylation, piperazine ring substitution and cleavage, and quinoline ring cleavage (Yang Y. et al., 2022). *Labrys portucalensis*, an alfa-Proteobacteia, also degrades fluoroquinolones (Amorim et al., 2014). Bacterial consortia could be more effective in antibiotic degradation; an ensemble of *Acetobacterium, Desulfovibrio, Desulfobulbus*, Peptococcaceae, *Lentimicrobium*, and *Petrimonas* might contribute to trimethoprim degradation in anaerobic conditions (Liang et al., 2019). In fact, consortia have been constructed on the bases of their high production of oxidases to increase biotransformation of antibiotics (Xu et al., 2022). Soil bacterial consortia efficiently degrade sulfonamides (Islas-Espinoza et al., 2012). Complex bacterial communities can be highly effective in antibiotic biodegradation, as has been described in the case of a consortium of Gamma, Beta-Proteobacteria, and Bacteroidetes degrading ciprofloxacin by deamination, hydroxylation, defluorination, and dealkylation (Liao et al., 2016). In this process, coupled with photocatalysis, Proteobacteria are particularly critical (Li et al., 2021).

## Planktonic microalgae and antibiotic biodegradation

Microalgae are prokaryotic and eukaryotic micro-organisms that can fix organic (autotrophic) and inorganic (heterotrophic) carbon. Cyanobacteria is probably the most common prokaryotic microalgae (Leng et al., 2020), and it significantly contributes to antibiotic removal *via* a process involving (as it was shown for tetracycline) biosorption and photodegradation (Pan et al., 2021; Wei et al., 2021). Eukaryotic microalgae include diatoms and green algae. Diatoms produce hydrogen peroxide (H_2_O_2_), which modifies and detoxifies complex organic molecules including antibiotics. A key mechanism in this process is the bio-Fenton reaction, which degrades hydrogen peroxide in the presence of iron particles, giving rise to the degradation of antibiotics, as has been shown with tetracycline (Pariyarath et al., 2021). Planktonic green algae can also degrade antibiotics. Early studies on antibiotics in the environment showed that green algae (genus *Nitella*) absorbed beta-lactams, phenicols, and aminoglycosides (Pramer, 1955). *Scenedesmus obliquus* is a frequent alga found in fresh and brackish water, particularly under conditions of anthropogenic pollution (Phinyo et al., 2017). It can degrade fluoroquinolones (such as levofloxacin) using a metabolic degrading pathway including cellular biocatalytic reactions including decarboxylation, demethylation, dihydroxylation, side chain breakdown, and ring cleavage (Xiong et al., 2017). The rate of antibiotic biodegradation (dissipation percentage) is variable among microalgae and various types of antibiotics. *Selenastrum capricornutum and Chlorella vulgaris* more efficiently degrade macrolides and fluoroquinolones than sulphonamides, which are better degraded by *Scenedesmus quadricauda* and *Haematococcus pluvialis* (Kiki et al., 2020). Microalgae communities with other microorganisms, such as filamentous fungi, could have synergistic effects in antibiotic detoxification (Leng et al., 2020).

## Fungi and degradation of antibiotics

Fungi belonging to the Basidiomycota, Ascomycota, and Mucoromycotina (formerly Zygomicota) subphyla can remove and transform antibiotic molecules. Ciprofloxacin is detoxified by conjugation with formyl, vinyl, or acetyl groups, or by hydroxylation or polymerization (Olicón-Hernández et al., 2017). *Aspergillus* and *Penicillium* appear to decrease the amount of ciprofloxacin in soil, but the underlying mechanism has not been elucidated; *Trichoderma* produce ciprofloxacin-conjugated inactive compounds when incubated with fluoroquinolones. *Mucoromycotina incertae* sedis (formerly Zygomycota) are also able to detoxify fluoroquinolones in a process involving N-oxidation, N-dealkylation, and N-acetylation. White-rot fungi (as the Basidiomycota *Pleurotus eryngii* or *Trametes versicolor*), widespread in nature due to their capability to degrade ubiquitous lignin, induce the production of extracellular low-molecular-weight extracellular oxidants, including oxygen-free radicals, mainly hydroxyl radicals, and lipid peroxidation radicals activating O_2_ in the environment and removing pollutants (Gómez-Toribio et al., 2009), most probably also antibiotic molecules.

## Plant removal and degradation of antibiotics

Independently from antibiotic adsorption to plant residues (Balarak et al., 2017), living plants may absorb a variety of antibiotics present in the soil, including anthropogenic quinolones (Eggen et al., 2011) This process is highly antibiotic dependent; for example, absorption is high for tetracyclines and low for macrolides (Kumar et al., 2005). Also, the type of plant determines absorption; oxytetracycline is accumulated in radish roots but not in lettuce leaves (Matamoros et al., 2022). Macrophytes such as duckweed and water fern absorb antibiotics by the roots and detoxify them by oxidation, conjugation, and storage in the plant (Maldonado et al., 2022). One example of such degradation is duckweed *Spirodela polyrhiza*, which degrades fluoroquinolones (Singh et al., 2019).

## Antibiotic sequestration and inactivation by living cells and the necrobiome

After cell death, cellular components can remain for extended periods of time in the soil or water. Many antibiotics, particularly macrolides, lincosamides, fluoroquinolones, tetracyclines, rifamycins, chloramphenicol, trimethoprim, and sulfonamides, and also beta-lactams to a lesser extent, enter eukaryotic cells where they are sequestered and inactivated. Interestingly, extracellular antibiotics have more activity than intracellular ones, although some of them accumulate intracellularly and reach high concentrations. One reason for the reduced activity of intracellular antibiotics is a presumed “impairment of the expression of antibiotic activity inside the cells” (van Bambeke et al., 2006). This field is important but poorly explored, and we know from human clinical trials that a renal dipeptidase, dehydropeptidase-I, can hydrolyze imipenem and other carbapenems (Birnbaum et al., 1985). Also, human and mammal liver microsomes (mimicking the activity of the endoplasmic reticulum) are able to biotransform fluoroquinolones, lincosamides, fluconazole, gentamicin, metronidazole, oxazolidinones, and even beta-lactams (Wynalda et al., 2000; Szultka et al., 2014; Szultka and Buszewski, 2016). Whether these results apply to other eukaryotic microsomes (including algae, fungi, plants, small animals, and protozoa) is not yet known. Nevertheless, an antioxidant defense mechanism is activated and glutathione S-transferase activity is significantly increased in aquatic plants such as *Azolla caroliniana* and *Taxiphyllum barbieri* exposed to tetracycline (Vilvert et al., 2017). Glutathione S-transferases, present in bacteria, fungi, plants, and animals inhibit beta-lactams sulfathiazole and tetracycline (Al-Mohaimeed et al., 2022).

Many antibiotics can be ultimately inactivated in matrixes constituted by massive amounts of dead bacteria (eventually killed by the antibiotics themselves; Hunt et al., 1987; Podlesek et al., 2016). This adsorption/detoxification of antibiotics by dead bacterial cells might be common in natural environments (Smakman and Hall, 2022). Envelopes of dead bacteria (such as lipopolysaccharide) and probably proteins (as in the case of “inoculum effect”) might bind to antibiotics (Peterson et al., 1985; Corona and Martínez, 2013). The same is possible with free DNA or RNA ribosomal fragments. Both aminoglycosides and beta-lactams can bind to the DNA helix *via* a minor groove binding model (Arya, 2005; Shahabadi and Hashempour, 2019).

## Anthropogenic environmental pollution and antibiotic detoxification

This review is oriented toward antibiotic “natural detoxification” in wild environments. However, human activities increasingly contribute to the composition of the Earth global environment. Most clinically-used antibiotics are released in areas close to densely human-populated patches, where also farming, agricultural, and industrial activities polluting the natural environment takes place. As stated before, metals are important agents in the detoxification of antibiotics, mostly involving oxidation and super-oxidation processes. Heavy (significant) metals pollution, involving lead, cadmium, chromium, mercury, or arsenic, and also iron, copper, cobalt, and silver are released from metal processing and smelting, chemical and manufacturing activities, factory emissions, and sewage irrigation (Yang et al., 2018). Even if metals might detoxify antibiotics, they also have antibacterial activities, frequently synergistic with clinical antimicrobial agents, and antibiotic-resistance genes are frequently found in antibiotic resistant bacteria, contributing to the evolutionary biology of these organisms (Baquero et al., 2021). Thus detoxification might be compensated by an enhanced antimicrobial effect, resulting in a stronger selection. Oil–water interfaces might influence antibiotic degradation (Basáez and Vanýsek, 1999) but also contribute to bacterial aggregations, as marine bacteria in oil spill (Ahmadzadegan et al., 2019), so that selection for antibiotic resistance effectively occurs (Shen et al., 2020). Water chlorination (partially?) detoxifies some antibiotics as azithromycin or fluoroquinolones (Jaén-Gil et al., 2020) but eventually have additive or synergistic effects with these drugs. The result is a decrease the bacterial count, which not excludes increased selection of resistance; in any case, this field has been scarcely explored. Bacteria from minerally-fertilized soils and crops reduce their content in antimicrobial resistance genes (Sanz et al., 2022), suggesting that chemical compounds, such as ammonium, sodium, or potassium sulfates, or superphosphates might reduce the selective effect of environmental antibiotics. Organic fertilizers, as pig manure and sewage sludge contain bacterial consortia able to detoxify antibiotics (see above), but it is to note that this effect could be compensated by the heavier pollution with antibiotic molecules originated in abusive use of antibiotics in humans and animals (Dong et al., 2021). Industrial composting (organic matter recycling) removes antibiotics and alters the local microbial ecology (Chen et al., 2021). Pollution by anthropogenic microplastics, is another aspect of anthropogenic pollution. Microplastics, small (less than 5 mm in length) fragments of any type of plastic, which also adsorb/detoxify antibiotics but also adhere bacterial cells and therefore contribute to the selection of resistance (Peng et al., 2022; Wang et al., 2022).

## The process of antibiotic adsorption, desorption, and inactivation and the evolution of antimicrobial resistance in microbiotic particles

**Figure 1:**
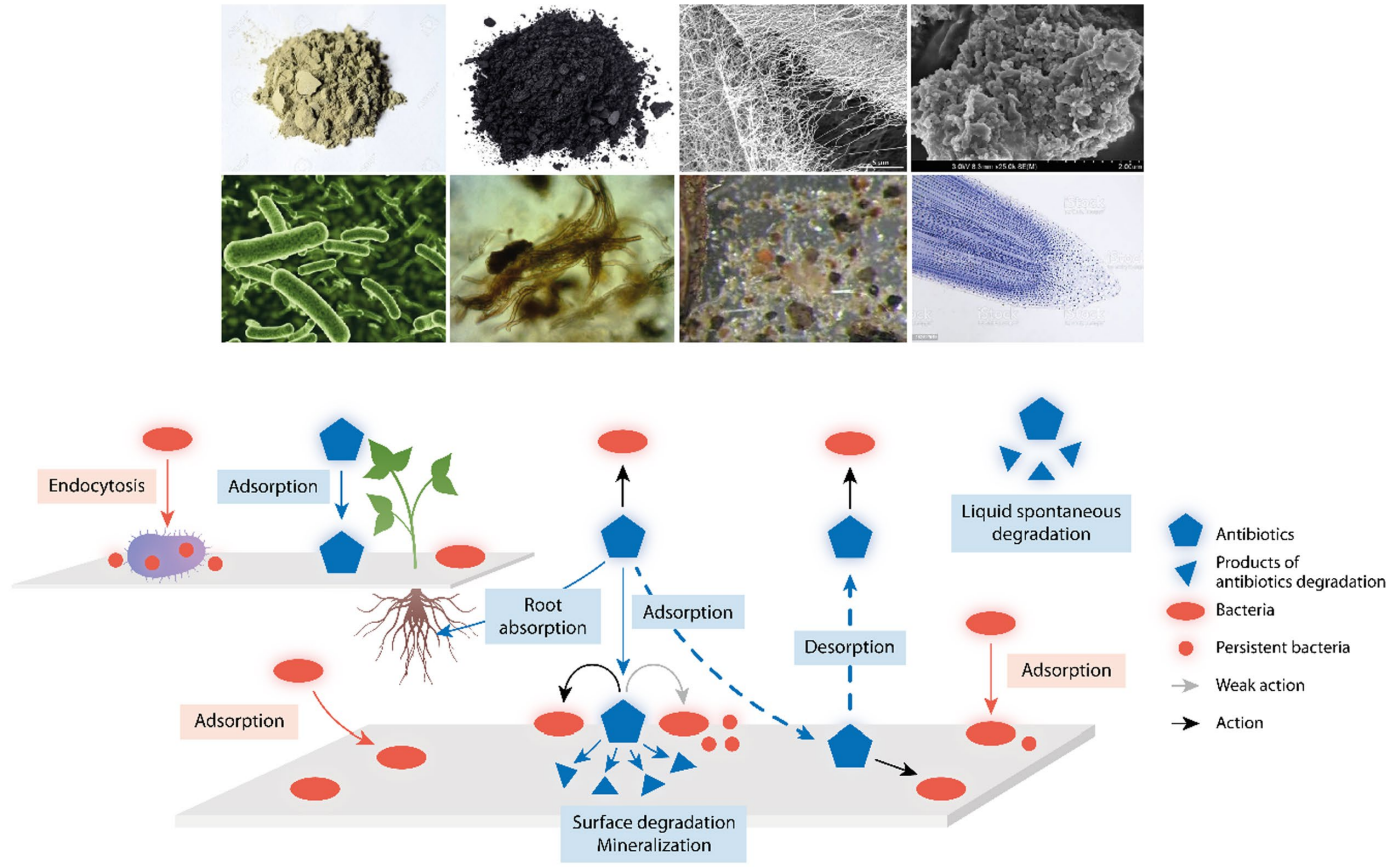
Environmental components influencing antibiotic action and detoxification. Up in the figure, examples of environmental components where antibiotics and bacterial cells might attach; from up left to down right, clay, charcoal, cellulose fibers, natural organic matter, bacteria, fungi, microfauna, and tip of plant roots. Down in the figure, a schema with a reactive surface from the above components where antibiotics (blue pentagons) and bacteria (red ovals) might attach; antibiotics can act (black arrows) or only weakly act (gray arrow) on sessile or attached bacteria; small blue triangles are the products of antibiotic degradation; and small red circles are persistent bacteria, phenotypically resistant to antibiotic action. The central broken arrows illustrate the key process of absorption/desorption of antibiotics in environmental surfaces, determining the PK/PD of these drugs, and therefore, the local selection of antibiotic resistant bacteria. Up left, a reactive surface illustrating the capture of bacteria by protozoa (as ciliates) favoring the release of bacterial pellets with antibiotic-persistent organisms, and the absorptive process of antibiotics by plant roots.

Deactivation of antibiotics in the environment should be beneficial for reducing selection and the environmental reservoir of antibiotic resistant bacteria. However, many mineral and biotic biomolecules deactivating antimicrobial agents are also part of soil and water particles that attach bacterial cells and in some cases are of anthropogenic origin (Baquero et al., 2022; Cydzik-Kwiatkowska et al., 2022). The key question is whether the adhesion of antibiotic molecules to these particles concentrates the antimicrobial agent, so that even if deactivation takes place, there could be enough selective power to select antibiotic resistant co-adhered cells. For example, if clays are able to remove antibiotics from the environment, high antibiotic concentrations would be found on the surface of clay particles, thereby contributing to the local selection of antibiotic resistant microorganisms (Lv et al., 2019). What we really need is to know better the PK/PD of antibiotics on environmental surfaces where bacteria might attach; in particular, if attached bacteria are phenotypically resistant to antibiotics, and how much of this effect is due to the local enrichment in persistent cells (because of the superoxide’s action). We cannot discard an antibiotic action on attached cells, but that should depend on the rates of absorption and desorption of the different antibiotics (Figure 1) and the local bacterial growth rates. The local microecological conditions as light and water availability, temperature, osmolarity, and pH are expected to modify such kinetics.

## Natural and anthropogenic antibiotic detoxification: A One health multifaceted process requiring integrated research approaches

Absorption, photolysis, hydrolysis, cation-binding, adsorption, bioaccumulation, and biodegradation simultaneously contribute to the removal of antibiotics from the environment (Xiong et al., 2017). Most probably, the mechanisms of antibiotic removal that we have reviewed work in combination, perhaps in synergistic detoxification, as has been observed in pollution control studies. For example, the combination of microalgae with irradiation and oxidation treatment favors antibiotic degradation (Leng et al., 2020). The increasingly complex anthropogenic influence on the environment, which releases and removes both antibiotics and antibiotic-resistant bacteria, is certain to influence the entire kinetics of antimicrobial drugs in the microbiosphere. However, current information on the effects of antibiotic detoxification in the environment is still fragmentary and a global, and an integrated and ecological view on the elements contributing to this process is needed. For example, earthworms, which change the exposure of soil organisms to ciprofloxacin, result in a much higher mineralization rate of antibiotics and illustrate the complexity of predicting the antibiotic detoxification processes (Mougin et al., 2013). We are still lacking highly efficient and comprehensive analytic procedures to dissect and quantify the chemical and biological composition of specific soil or water environments that are exposed to intensive antibiotic pollution. Such integrated analyses could help measure the hazard of antibiotic release in particular places at defined time-periods. Among the required parameters, soil volumetric water content (Briciu-Burghina et al., 2022), the total organic matter (Yang C. et al., 2022), or the “amount of surface” in the soil (for instance, total surface of clay particles) can be calculated and expressed as “specific surface area,” the surface area/unit mass of the dry soil with units of m^2^/g (Cerrato and Lutenegger, 2002). Antibiotics with a high adsorption potential on clay or organic matter tend to accumulate and persist in this matrix, whereas those having a lower adsorption potential are easily transported to the aquatic environment. More study of the microbial ecology of antibiotic molecules is needed, given that there are potential gaps between the analytical results obtained in the lab and the in the environment (Polianciuc et al., 2020). Pedological sciences should approach microbiology to match soil classifications with local environmental pharmacokinetics and the pharmacodynamics of antimicrobial agents. Techniques able to measure the physical and chemical adsorption of antibiotic and bacterial molecules taking up by the different types of surfaces (with different energy distributions; Webb, 2003) should be developed to reach such a goal. Everything on Earth is intertwined, and the goal of One health (Hernando-Amado et al., 2019) is fully dependent on the geochemical and biological structure of the particular environments and requires an interdisciplinary effort (Brantley et al., 2007). We need to progress toward the definition of “local bio-geo-chemical reactive profiles,” so that we can understand the reactive transport (Carrera et al., 2022) of antibiotic molecules. That step will be indispensable in shaping appropriate environmental “One health” interventions to reduce microbial resistance to antimicrobial agents.

## Author contributions

FB conceived the topic, discussed in depth with TC and J-LM, wrote the manuscript, and prepared the figure. All authors contributed to the article and approved the submitted version.

## Funding

This work, developed in the Ecoevobiome lab of the Department of Microbiology of the Ramón y Cajal Hospital (IRYCIS; http://www.ecoevobiome.org/), was supported by the European Union—Next Generation EU (MISTAR Project—AC21_2/00041) and the Instituto de Salud Carlos III (ISCIII) of Spain, also funded by ISCIII through project PI21/02027, co-financed by the European Union, and by the Spanish Network for Research in Infectious Diseases, CIBERINFEC (CB21/13/00084), and CIBERESP (CB06/02/0053).

## Conflict of interest

The authors declare that the research was conducted in the absence of any commercial or financial relationships that could be construed as a potential conflict of interest.

## Publisher’s note

All claims expressed in this article are solely those of the authors and do not necessarily represent those of their affiliated organizations, or those of the publisher, the editors and the reviewers. Any product that may be evaluated in this article, or claim that may be made by its manufacturer, is not guaranteed or endorsed by the publisher.
